# Evaluation of circulating tumor DNA by electropherogram analysis and methylome profiling in high-risk neuroblastomas

**DOI:** 10.3389/fonc.2023.1037342

**Published:** 2023-05-12

**Authors:** Eva María Trinidad, Antonio Juan-Ribelles, Giulia Pisano, Victoria Castel, Adela Cañete, Marta Gut, Simon Heath, Jaime Font de Mora

**Affiliations:** ^1^Laboratory of Cellular and Molecular Biology, Health Research Institute Hospital La Fe, Valencia, Spain; ^2^Clinical and Translational Research in Cancer, Health Research Institute Hospital La Fe, Valencia, Spain; ^3^Pediatric Oncology Unit, La Fe University Hospital, Valencia, Spain; ^4^School of Medicine, University of Valencia, Valencia, Spain; ^5^National Center for Genomic Analysis – Centre for Genomic Regulation (CNAG-CRG), Centre for Genomic Regulation (CRG), Barcelona Institute of Science and Technology (BIST), Universitat Pompeu Fabra (UPF), Barcelona, Spain

**Keywords:** liquid biopsy, high-risk neuroblastoma, DNA mehtylation, ctDNA / normal cfDNA ratio, enzymatic methyl-sequencing (EM-seq)

## Abstract

**Background:**

Liquid biopsy has emerged as a promising, non-invasive diagnostic approach in oncology because the analysis of circulating tumor DNA (ctDNA) reflects the precise status of the disease at diagnosis, progression, and response to treatment. DNA methylation profiling is also a potential solution for sensitive and specific detection of many cancers. The combination of both approaches, DNA methylation analysis from ctDNA, provides an extremely useful and minimally invasive tool with high relevance in patients with childhood cancer. Neuroblastoma is an extracranial solid tumor most common in children and responsible for up to 15% of cancer-related deaths. This high death rate has prompted the scientific community to search for new therapeutic targets. DNA methylation also offers a new source for identifying these molecules. However, the limited blood sample size which can be obtained from children with cancer and the fact that ctDNA content may occasionally be diluted by non-tumor cell-free DNA (cfDNA) complicate optimal quantities of material for high-throughput sequencing studies.

**Methods:**

In this article, we present an improved method for ctDNA methylome studies of blood-derived plasma from high-risk neuroblastoma patients. We assessed the electropherogram profiles of ctDNA-containing samples suitable for methylome studies, using 10 ng of plasma-derived ctDNA from 126 samples of 86 high-risk neuroblastoma patients, and evaluated several bioinformatic approaches to analyze DNA methylation sequencing data.

**Results:**

We demonstrated that enzymatic methyl-sequencing (EM-seq) outperformed bisulfite conversion-based method, based on the lower proportion of PCR duplicates and the higher percentage of unique mapping reads, mean coverage, and genome coverage. The analysis of the electropherogram profiles revealed the presence of nucleosomal multimers, and occasionally high molecular weight DNA. We established that 10% content of the mono-nucleosomal peak is sufficient ctDNA for successful detection of copy number variations and methylation profiles. Quantification of mono-nucleosomal peak also showed that samples at diagnosis contained a higher amount of ctDNA than relapse samples.

**Conclusions:**

Our results refine the use of electropherogram profiles to optimize sample selection for subsequent high-throughput analysis and support the use of liquid biopsy followed by enzymatic conversion of unmethylated cysteines to assess the methylomes of neuroblastoma patients.

## Introduction

Neuroblastoma is the most common extracranial solid tumor in children and responsible for up to 15% of cancer-related deaths (1). These tumors are heterogeneous with extreme clinical variability ranging from spontaneous regression to rapid progression and metastasis. Risk markers (histology, age, *MYCN* amplification, INRG stage, ploidy status, and 11q aberration) are used to divide patients into three categories: low, moderate, and high (2). Low and intermediate groups show greater than 90% five-year survival rates, whereas the survival of the high-risk group remains around 40%, which decreases to 10% upon relapse (3). Approximately half of all newly diagnosed neuroblastomas are designated high-risk for relapse. Therefore, there is an urgent need to develop novel treatment targets to improve the survival rate of high-risk neuroblastoma patients. Primary neuroblastoma tumors have been shown to contain a low number of coding mutations (4) suggesting that either they have very few potent oncogene/tumor suppressor drivers or that there are alternative mechanisms underlying tumorigenesis. Some high-risk neuroblastoma tumors display somatic amplifications/mutations in oncogene drivers, such as *MYCN*, *ALK*, *PTPN11*, *ATRX* and *NRAS* (5, 6). However, other high-risk neuroblastoma tumors do not harbor either *MYCN* amplification or mutations in other known tumor drivers, suggesting that perhaps epigenetic changes, such as DNA methylation, are involved in the tumorigenesis process. Large-scale genome sequencing projects have shown the importance of the epigenome to neuroblastoma (7). Recently, we have found a DNA methylation profile in high-risk neuroblastoma that discriminates between the main groups within high-risk: *MYCN*-*amplified* and 11q-deleted. Moreover, we demonstrated that some methylated genes, including CCR7 and CSF1R, have prognostic value in 11q- deleted subgroup (8).

Liquid biopsy has emerged as a potentially outstanding tool for spatiotemporal studies of cancer dynamics as well as an accurate monitor of disease (9, 10). In neuroblastoma patients, blood-derived plasma cell-free DNA (cfDNA) has shown its feasibility and accurate representation of the tumor regarding copy number variations (CNVs) (11) and exon mutations (12). cfDNA is typically found as double-stranded fragments of approximately 150 - 200 base pairs in length, corresponding to the unit size of nucleosome (13). cfDNA is released into the bloodstream by cellular processes involving apoptosis, necrosis and secretion (14). In healthy populations, it is found at a very low concentration, often less than 10 ng per ml of plasma (15), but under some circumstances this amount could increase, including trauma, myocardial infarction, stroke, chronic diseases and cancer. When this cfDNA is released by tumor cells, it is termed circulating tumor DNA (ctDNA) and its levels are related to stage and tumor burden, increasing up to 50 times that of normal levels. Consistent with this notion, a favorable response to cancer treatment is associated with a rapid decrease in blood-based cfDNA concentration in patients with high-risk neuroblastoma (16).

Thus, ctDNA fraction has become a surrogate marker for the staging, prognosis, monitoring response and minimal residual disease, and identification of acquired drug-resistance mechanisms (17). Notably, the distribution patterns of different DNA size occur independently of disease status (16), suggesting that cellular processes involved in DNA fragmentation are unique to the tumor biology in each patient and not to pathological conditions of the disease. The proportion ctDNA/cfDNA is highly variable, ranging from <0.05% (18) to 90% (19), so adequately discriminating between one and the other and determining the suitable threshold is essential to be successful in its analysis. Elevated ctDNA fraction has been reported to be relevant for adequate tumor profiling and for the identification of genetic alterations across cancer types (20). Elevated ctDNA fraction was also found reliable for identifying targetable kinase fusions across cancer types (21). In an ovarian cancer study, whole genomic sequencing-based CNVs were detected in the tumor with a median of 50% of genome altered fraction, but in the ctDNA with a median fraction of 12.7% (22). This plasma genome altered fraction was associated with progression-free survival (PFS), supporting the clinical value of ctDNA fraction. One interesting study on the correlation of metastatic location and ctDNA fraction in colon cancer revealed that, patients with lung-only and peritoneum-only metastatic disease, had significantly lower levels of ctDNA, suggesting decreased ctDNA released and clinical sensitivity depending on sites of metastasis (23). In non-small cell lung cancer patients, higher ctDNA fraction and detection after initial treatment was associated with shorter PFS, hence identifying patients who may benefit from further therapeutic intervention (24). Importantly, a retrospective analysis across tumor types revealed that ctDNA quantity varied significantly based on patient age, sex, stage, and tumor type, explaining why certain liquid biopsy specimens are more likely to fail sequencing or provide clinically meaningful results (25). Genomic ctDNA profiling has also demonstrated its value in neuroblastoma, showing clonal evolution dynamics in somatic alterations that increase at relapse and therefore, pointing out its use for targeted therapies (12). Recently, ctDNA was reported to be prevalent in children with high-risk neuroblastoma and valuable for follow-up during neuroblastoma treatment (26).

DNA methylation profiling has become a promising approach for sensitive and specific detection of many cancers. Bisulfite conversion of unmethylated CpGs has been the standard method for methylation profiling. Reduced Representation Bisulfite Sequencing (RRBS) is a good method for DNA methylation studies, covering about 3 million CpG sites, with an affordable cost (27). However, current techniques for capturing CpG-rich regions may result in loss of some functionally relevant information. Also, RRBS covers approximately 20% of the total CpG islands in the genome (28) and only 60% of the promoter regions (29). Additionally, this method excludes genes lacking or with a distant CCGG motif. Therefore, use of RRBS could result in failure to obtain complete and relevant information due to the lower coverage of this technique. Another effective approach for targeted methylome studies is the heat enrichment of CpG-rich regions for bisulfite sequencing (Heatrich-BS) (30). Heatrich-BS allows methylation profiling in highly informative regions, but it fails to capture information on single CpG or small CpG islands. In addition, bisulfite causes DNA degradation, resulting in the loss of information limited in samples with very small amounts of ctDNA. Therefore, several bisulfite conversion-free methods have been developed and optimized for the detection of cfDNA methylation (27). Several bisulfite genomic sequencing studies across neuroblastoma risk groups reported differential methylation profiles of genes that are associated to prognosis (*SCNN1A*, *PRKCDBP*, *KRT19*; *HIST1H3C*, *GNAS*, and a 58 gene signature) (31–33).

Recent studies with immunoprecipitation of cell-free methylated DNA coupled with next-generation sequencing (cfMeDIP-seq) have shown this an effective approach for the analysis of cfDNA methylome from minute quantities of cfDNA (34, 35). However, this technical approach may not detect valuable information in hypomethylated regions relevant to cancer progression. cfDNA fragmentation patterns contain important molecular information linked to tissues of origin and gene expression inference. Recent studies on fragmentomics-based methylation analysis (FRAGMA) have shown the feasibility of using cfDNA cleavage patterns to deduce CpG methylation at single CpG resolution using a deep learning algorithm (36). Moreover, a recent study demonstrates that libraries made using the Enzymatic Methyl-seq (EM-seq), another bisulfite-free method, outperformed bisulfite-converted libraries in all specific measures examined (coverage, duplication, sensitivity, etc.) (37).

In the present article, we use ctDNA from high-risk neuroblastoma patients to compare two conversion methods of unmethylated cysteine, bisulfite and enzymatic conversion, for whole methylome sequencing studies. We also analyze the tumor fraction (ctDNA/cfDNA) content at diagnosis and relapse to establish the suitable threshold for optimal analysis of tumor methylome profile. Several key points for the successful methylome study of high-risk neuroblastoma are indicated in our results.

## Material and methods

### Patients and simples

A retrospective study was performed in a primary cohort of 86 patients diagnosed with primary high-risk neuroblastoma who underwent treatment between 2007 and 2019. 79 patients were classified in M stage and 7 in L2 stage, with mean age of 42 months at diagnosis. 43 relapsed (the mean time to relapse was 17.5 months), of whom 41 samples were collected. These cases were collected from the archives of Hospital La Fe in Valencia. Clinical characteristics for each patient are summarized in **Table 1**. Around 1 ml of blood was obtained from neuroblastoma patients. Written informed consent was signed by the patients; when not possible (dead or unreachable patients), the study material was used after decoding in accordance with Spanish law and with the approval of the Institutional Review Board. All procedures were done in accordance with the Helsinki declaration.

**Table 1:** Clinical characteristics of the high-risk neuroblastoma patients included in the study.

	INRG		
Characteristics	M	L2	Total
Number of patients			
	79	7	86
Age at diagnostic in months			
Median	42.03	23.8	41.6
Range	(2.67 - 185.8)	(9.5 - 74.1)	(2.67 - 185.8)
Sex			
Female	32	4	36
Male	47	3	50
Relapse			
Yes (%)	40 (50.1%)	3 (43%)	43 (50%)
No (%)	39 (49.4)	4 (57%)	43 (50%)
Median time to relapse (month)	17.5	20.90	17.5

### Isolation quantification and analysis of ctDNA

Whole blood samples were collected in PAXgene tubes and kept at room temperature for no more than 24 hours before plasma was collected by centrifugation two consecutive times for 15 minutes at 2000xg. Plasma was stored at -80°C until the moment of use. DNA was isolated from 1 ml of blood-derived plasma with QIAamp Circulating Nucleic Acid Kit (QIAGEN). Quantity and quality of isolated ctDNA was determined by using D1000 ScreenTape assay in Agilent 4200 TapeStation. We have also used this method to quantify DNA, focusing on mono-nucleosomal fragments around 100-200 base pairs, but other larger nucleosome sizes were also considered. The median of ctDNA from diagnosis and relapse samples, collected from 1 ml of blood, was 24.2 ng. Quantity of double-stranded DNA was also determined with Qubit (Thermofisher).

### Bisulfite conversion-based method

This method is based on the conversion of unmethylated cytosine to uracil, while methylated cytosines remained unchanged. The QIAseq Methyl Library Kit (QIAseq) was used to prepare libraries from bisulfite-treated DNA samples for whole genome methylation studies using Illumina platforms. QIAseq Methyl Library kit allows DNA inputs as low as 100 pg to deliver high-quality, high-yield libraries for use with Illumina platforms. Libraries were constructed with 10 ng or 20 ng of DNA, as indicated, following the manufacturer recommendations.

### Enzymatic methyl-sequencing

NEBNext Enzymatic Methyl-seq (EM-seq) was used for the identification of 5-methylcytosine and 5-hydroxymethylcytosine. 10 or 20 ng starting material of cfDNA, as indicated, were used on a two-step conversion of the cytosines. In the first step TET2 oxidation of 5-methylcytosine and oxidation enhancer of 5-hydroxymethylcytosine provided protection to the modified cytosines from conversion by APOBEC deamination, performed in the second reaction. Ultimately, cytosines are sequenced as thymines and 5-methylcytosines or 5-hydroxymethylcytosines are sequenced as cytosines. The final 8 cycles of PCR library amplification were carried out using the NEB Unique dual index primer pairs (NEBNext multiplex oligos for Illumina).

### Sequencing and data analysis of ctDNA methylation and CNV assays

The libraries were sequenced on NovaSeq 6000 (Illumina) in paired-end with a read length of 2x151 bp according to the manufacturer’s protocol for dual indexing. Image analysis, base calling and quality scoring of the run are processed using the manufacturer’s software Real Time Analysis (RTA 3.3.3) and followed by generation of FASTQ sequence files.

The EM-seq reads were processed using the gemBS pipeline v4.03 using as reference GRCh38. Reads with MAPQ scores < 20 and read pairs mapping to the same start and end points on the genome were filtered out after the alignment step. The first 5 bases from each read were trimmed before the variant and methylation calling step to avoid artifacts due to end repair. For each sample, CpG sites were selected where both bases were called with a Phred score of at least 20, corresponding to an estimated genotype error level of <=1%, and where the total number of reads informative for methylation from both strands combined was >=6. Sites with >500x coverage depth were excluded to avoid centromeric/telomeric repetitive regions. Sequencing depth for the analysis of CNV was calculated using samtools v1.15. Liquidhope cohort data is available at Geo database (GSE221317; https://www.ncbi.nlm.nih.gov/geo/query/acc.cgi?acc=GSE221317).

## Results

### EM-seq was more efficient than bisulfite conversion for whole genome methylome sequencing

**Figure 1:**
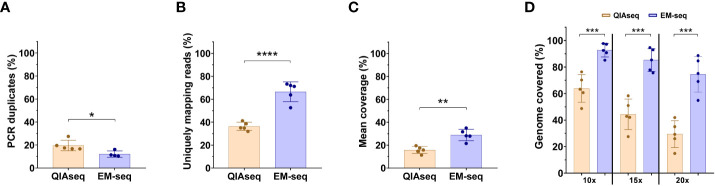
NEBNext Enzymatic Methyl-Sequence method outperforms the WGBS-QIAsequence method. The comparison of the library yield that passed the Illumina filter for WGBS-QIAsequence (QIAseq) and NEBNext Enzymatic Methyl-Sequence (EM-seq) libraries. **(A)** Percentage of PCR duplicates. **(B)** Percentage of uniquely mapping reads. **(C)** Mean of coverage. **(D)** Comparison of the percentage of the mappable reference with 10, 15 or 20X coverage using QIAseq and NEB Next-EM-seq methodologies. Five-paired samples were used to compare both methods. Each dot represents a neuroblastoma sample. Statistical differences were tested by two-tailed Student t-test (p< 0.05 *, p<0.01 **, p<0.001 ***, p<0.0001 ****), comparing QIAseq and EM-seq method.

To gain novel information regarding methylome studies and DNA methylation biomarkers relevant for high-risk neuroblastoma, we assessed the performance of EM-seq *vs*. bisulfite conversion methods for implementing methylome profiling in liquid biopsies (**Figure 1**; **Supplementary Figure S1**). Results from both methodologies indicated an optimal library size and integrity of the ctDNA obtained. However, we obtained higher library concentration (ng/µL) with the EM-seq approach (**Table 2**). Of note, we also compared the use of 10 ng versus 20 ng of DNA, with similar results in terms of the library concentration (p = 0.57 for QIAseq; p = 0.55 for EM-seq), but again, so we used 10 ng of DNA. The comparison between the QIAseq and the EM-seq approach, performed five to six times higher in the DNA library content for the EM-seq method (**Table 2**).

**Table 2:** Library and size comparison between QIAseq and EM-seq conversion methods.

			QIAseq		EM-seq	
Gender	Stage	Imput ctDNA (ng)	Library [ng/ul]	Size (bp)	Library [ng/ul]	Size (bp)
Male	M	20	6.26	326	11.26	304
Male	M	20	6.53	319	102.49	297
Female	M	10	7.7	327	35.79	301
Female	M	20	7.74	324	42.46	302
Male	L2	10	6.84	309	21.27	299

Five-paired samples were used to compare both methods in parallel.

Regarding the conversion efficiency, the QIAseq method does not include an internal quality control of conversion; the EM-seq does have this control. Therefore, we compared the conversion rate of unmethylated cytosines by focusing on non-CpG sites (assuming that most of these will be unmethylated). With the EM-seq method, we obtained 99.8% conversion efficiency using this metric, and with QIAseq method we obtained a conversion efficiency of 99%. The comparison of the library yield (Gb generated in the sequencing run) between QIAseq and EM-seq showed similar results (**Supplementary Figures S1A, B****)**. Both techniques generated similar amounts of sequencing data with no significant differences, referred to by either total millions of reads (**Supplementary Figure S1A**), or by Gigabases (**Supplementary Figure S1B**). Although the amount of sequencing data obtained was similar between the two methods, the EM-seq method showed significant differences in the rest of the parameters evaluated. The EM-seq method yielded a lower proportion of PCR duplicates, thus, reducing the possibility of false positive variant calls (**Figure 1A**). EM-seq also had a higher percentage of unique mapping reads (**Figure 1B**), offering a higher mean coverage (**Figure 1C**) and a higher genome coverage (**Figure 1D**). The percentages of the mappable references with at least 10x, 15x, or 20x coverage depth are highly uniform for the EM-seq and superior to those obtained with the QIAseq approach (**Figure 1D**). Thus, our results suggest that the EM-seq is more optimal than the QIAseq method for whole methylome studies.

### Electropherogram-based identification of samples with suitable ctDNA content

To determine the best technique for the study of ctDNA methylome from liquid biopsies, DNA was isolated from 1 ml of blood-derived plasma of 126 samples from 86 patients diagnosed with high-risk neuroblastoma, 40 paired samples at diagnosis and at relapse, 44 samples only at diagnosis and 2 samples only at relapse, (**Supplementary Table S1**). All samples were evaluated with D1000 ScreenTape to determine the quality and quantity of DNA (**Supplementary Table S1**). Although the electropherogram profile of cfDNA in pediatric tumors, including neuroblastoma, has its use and variability among different biofluids (16), its interpretation as potential reservoir of valuable tumor DNA remains elusive.

To further discriminate when a sample is sufficiently enriched with ctDNA, we combined electropherogram profiles with sequencing-based CNVs. Following the initial diagnosis, we also validated the presence of *MYCN* amplification or/and 11q deletion in each patient by analyzing sequence-based CNVs from ctDNA (**Supplementary Table S1**). In addition, we further evaluated the methylation profile of chromosomes, considering a ctDNA-containing sample when hypomethylation values dropped down to 0.25 or lower, resulting in sharp changes in the methylation profiles. Samples with no or highly diluted ctDNA barely showed sharp changes in their methylation profile, displaying a waviness profile ranging between 0.75 and 1 (**Supplementary Table S1**). *In vitro* enrichment of ctDNA was originally focused on mechanical isolation of mono-nucleosomal fragments (later on defined ctDNA with a median fragment length of 134–144 bp) to improve the ratio between ctDNA and cfDNA (38). In here we used a simplified approximative method calculating mono-nucleosomal/total cfDNA content as an estimate of ctDNA ratio (**Supplementary Table 1**).

**Figure 2:**
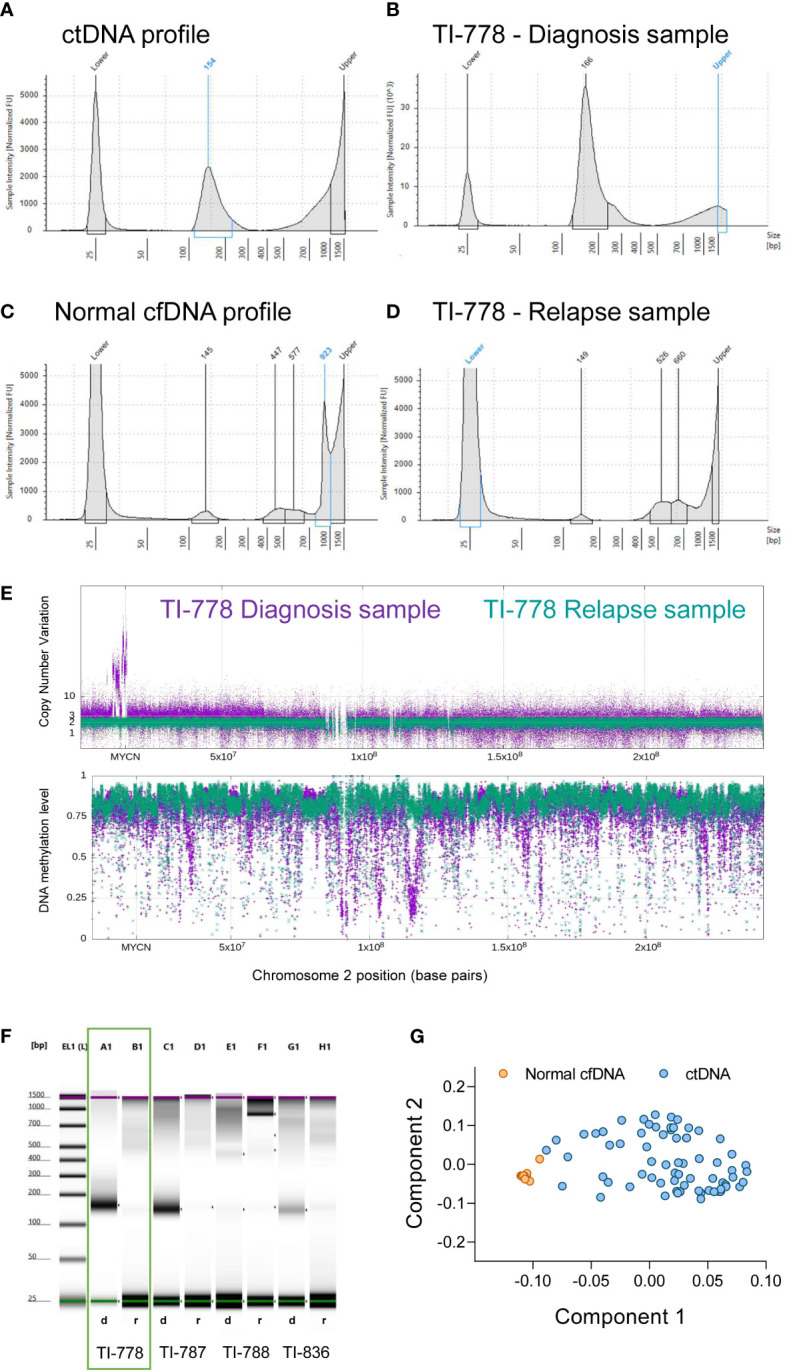
Electropherogram profiles retain relevant information to interpret ctDNA/cfDNA content in blood-derived plasma samples. **(A-D)** The characteristic profiles in TapeStation electropherograms (mono-nucleosomal peak and higher fragments) allow the discrimination between samples enriched in ctDNA **(A, B)** and normal cfDNA **(C, D)**. Electropherograms include two picks for the low and high molecular weights (25 and 1500 bp), as indicated, corresponding to the ladder controls used to estimate sizes. Electropherogram images of the paired samples in orange square (patient TI-778) are represented at diagnosis **(B)** and at relapse **(D)**. **(E)** Whole genome methylome analyses corroborated that sample TI-778 at diagnosis (in purple) contained valuable tumor DNA, while at relapse (in green) contained mostly normal DNA. **(F)** Representative electrophoretic runs in a D1000 ScreenTape assay of four paired samples at diagnosis d) and relapse r). **(G)** Principal components model that discriminates tumor DNA-enriched samples that cluster together (blue circles), from normal DNA-containing samples (orange circles).

Samples containing a mono-nucleosomal peak at around 100-200 base pairs, with or without a smaller peak at di-nucleosomal size and sometimes also at tri-nucleosomal size (**Figures 2A, B****)**, contained CNVs as detected by sequencing analysis. In addition, the profile of methylation averaged over 1kb bins for these samples and displayed extended regions of hypo-methylation with blocks of methylation extending over many kb with average methylation below 0.5 (**Figure 2E**, purple trace; **Supplementary Figure 2C**). In contrast, samples with low content of mono-nucleosomal peak and with high molecular weight DNA contamination, showed an absence of CNVs, with the average methylation profile remaining predominantly above 0.7, with no evidence of extended regions of hypo-methylation below 0.5 (**Figure 2E**, green trace; **Supplementary Figure 2D**). These samples without detectable ctDNA or with highly diluted ctDNA based on the absence of mono-nucleosomal peak, the absence of CNVs and by the absence of extended hypomethylation regions below 0.5 were referred to as normal cfDNA (**Figure 2C, D****)**. In addition to a rather small mono-nucleosomal peak, normal cfDNA may contain multi-nucleosome peaks with increasing DNA quantities (**Figure 2C**). Samples of disease-free neuroblastoma patients (at the end of treatment or during subsequent follow-up) lacked the mono-nucleosomal fragment (**Supplementary Figures S2A, B****)**, further supporting that the mono-nucleosomal fragment is an indicator of ctDNA content.

The sample for patient TI-778 at the time of diagnosis (purple, upper panel, **Figure 2E**), exhibited many copies at *MYCN* locus on chromosome 2, confirming the diagnosis with *MYCN* amplification, and depth variations in methylation profile (**bottom panel**, **Figure 2E**). However, the relapse profile from the same patient (depicted in green) did not exhibit *MYCN* amplification, nor variation of its methylation profile, suggesting that ctDNA content was low and highly diluted by normal cfDNA, further confirming our previous observations in the TapeStation profiles (**Figures 2B, D, F****)**. We evaluated the ctDNA content in samples based on principal component analysis (PCA) of the sequenced methylome in each sample (**Supplementary Table S1**). Analysis of the Component 1 and Component 2 showed that samples considered to contain normal DNA clustered together, whereas those samples containing significant amounts of ctDNA grouped distinctly from the normal samples (**Figure 2G**).

Electropherogram profiles were classified as tumor or normal based on their methylome and genomics profiles. Since the mono-nucleosomal peak is considered to be principally of tumor origin (38), we used the electropherogram profile in each sample to calculate the ratio mono-nucleosomal (100-200 pb)/total low molecular weight DNA (100-1000 pb) (**Supplementary Table S1**). We established a biased cut-off ratio of 10% and noted that it was successful in 95.1% of the cases studied; the prediction failed in only 4 out of 81 samples for which we had CNV and methylation profiles (**Supplementary Table S1**). For these 4 cases, we also evaluated Qubit quantification (**Table 3**). We observed that the Qubit quantification in these cases was much greater than the quantified peaks in the electropherogram. One possibility is that the limited range of sizes analyzed by D100 TapeStation do not cover the higher molecular weights of DNA released by cells that succumb by necrosis instead of apoptosis. These results suggest that the contribution to cfDNA by different cellular processes varies in each patient depending on their specific tumor conditions such as size, proliferation, dissemination and others. These results support that analysis of TapeStation electropherograms, combined with total DNA measurements by Qubit, may constitute a relevant tool for determining the presence of ctDNA in a sample.

**Table 3:** Qubit quantification in ctDNA-containing samples with reduced mono-nucleosomal peak.

Patient	Sampling time	Mono-nucleosomal/Total amount	Prediction based on ratio	PCA clustering	Qubit quantification (ng)
*TI-745*	Relapse	0.131	Tumor	Normal	815
*TI-913*	Relapse	0.059	Normal	Tumor	940
*TI-1017*	Diagnosis	0.066	Normal	Tumor	290
*TI-1041*	Diagnosis	0.044	Normal	Tumor	287.5

The comparisons of CNV and hypomethylation profiles, with TapeStation electropherogram profiles was also extended to evaluate the relative quantity of mono-nucleosomal peak as an estimate of the ctDNA content in the sample (**Supplementary Figures S2A-D**; **Supplementary Table S1**). The mono-nucleosomal peak was most abundant in most, but not all, ctDNA containing samples, but showed a low concentration when normal cfDNA was prevalent and was diluted with multi-nucleosomal peaks that increased in quantity with their size (**Supplementary Figure S2**; **Supplementary Table S1**).

### Relapse blood samples contains less ctDNA

**Figure 3:**
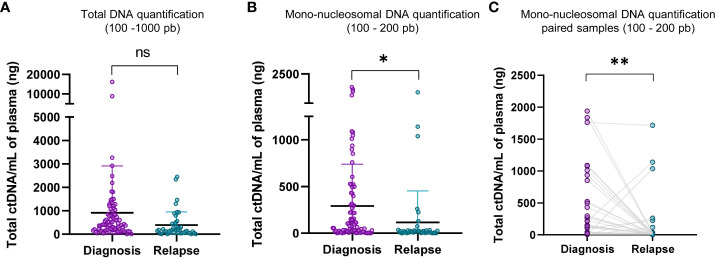
Mono-nucleosomal DNA is less abundant in relapse samples. **(A)** Comparison of total DNA quantifications of fragments within the range of D1000 ScreenTape assays (100 – 1000 bp) between diagnosis and relapse samples. **(B)** Comparison of mono-nucleosomal DNA quantifications (100 – 200 bp) between diagnosis and relapse samples. The quantification corresponding to nucleosomal fragment, 100 - 200 pb, to diagnosis and relapse in paired samples was shown in **(C)**. Statistical differences were tested by two-tailed Student t-test (ns, not significant p>0.05, p<0.05 *, p<0.01 **).

The quantification of the total cfDNA (100 – 1000 bp, TapeStation electropherogram) did not exhibit a significant statistical difference between diagnosis and relapse samples (**Figure 3A**). However, the quantification of mono-nucleosomal fragment size (100-200 bp) showed that samples at diagnosis contained a higher amount of ctDNA (**Figure 3B**). This increase was further magnified when considering paired diagnosis and relapse samples (**Figure 3C**). A representative image of TapeStation quantification is shown in **Figure 2F**, with 4 samples at diagnosis (A1, C1, E1 and G1 wells, **Figure 2F**) and their corresponding relapse samples (B1, D1, F1 and H1, **Figure 2F**), and whose mono-nucleosomal quantifications is indicated in **Supplementary Table S1**. The amount of ctDNA found was higher for samples at diagnosis than for samples at relapse (**Figure 3C**).

DNA quantification was compared at diagnosis and at relapse (see representative patient TI-778 in wells A1 and B1 in **Figure 2F**, orange square; quantification for all patients is shown in **Supplementary Table S1**). Electropherogram profile at diagnosis (**Figure 2B**) coincided with the expected profile for a sample containing valuable ctDNA (**Figure 2A**), with a major peak at mono-nucleosomal size and in some samples with bi- and tri-nucleosomal peaks that were reduced in quantity as the size of fragments increased. At relapse, samples more frequently showed a small mono-nucleosomal peak diluted by other multi-nucleosomal peaks (see representative patient TI-778 in **Figure 2E**). In parallel, sequencing data was used to estimate CNV and confirmed the original status for *MYCN* amplification, 11q deletion (**Supplementary Table S1**), or other segmental chromosomal aberrations (SCA). Those cases that did not have any SCA were considered as highly diluted with normal cfDNA, and their normal assessment was further confirmed by the reduced hypomethylation profiles derived from the sequencing (**Figure 2E**; **Supplementary Table S1**) and by the tumor-independent clustering in the PCA (**Figure 2G**; **Supplementary Table S1**). These distinct electropherogram profiles demonstrate that samples at relapse have a lower ctDNA content per ml of blood than samples taken at the time of diagnosis.

## Discussion

Analysis of ctDNA methylation has emerged as a promising tool with many potential clinical applications in the field of neuroblastoma (16). However, in attempting to apply this approach to pediatric patients, we previously encountered limitations with the ctDNA quantity and/or quality (ctDNA was present but significantly diluted with normal cfDNA) to perform methylation studies. These limitations arising from normal/tumor origin of cfDNA are relevant for making decisions regarding whether to submit a sample to subsequent techniques and analyses. Given the relevance of resolving these technical obstacles, we assessed two cytosine-conversion techniques for ctDNA methylome studies, and found that the EM-seq method performed better than the bisulfite technique.

Both RRBS and whole genome methyl-sequencing, allow the identification of pan-methylation status of cytosines with very low input amount of DNA, in contrast to array-based analysis that requires over 100 ng of DNA, an unattainable amount for liquid biopsy samples in most of pediatric cases. In our search to identify a suitable technique for whole genome methyl-sequencing study from limited amounts of ctDNA (10 ng), we evaluated two methods, the QIAseq, based on the standard genome bisulfite conversion, and EM-seq methods, based on two-step enzymatic conversion process. Our comparison between the different techniques indicates that, although conversion efficiency, library size and integrity obtained were optimal in both methodologies, we achieved higher library concentration, five to six times higher, with the EM-seq approach (**Table 2**). EM-seq also demonstrated better efficiency when we evaluated the proportion of PCR duplicates, the percentage of uniquely mapping reads, the mean coverage and genome covered (**Figures 1A-D**), supporting the use of EM-seq over QIAseq method for ctDNA-based methylome studies. In agreement with our results, comparisons between EM-seq and bisulfite methods using genomic DNA methylome analysis showed that EM-seq performed better for library and sequencing quality; the EM-seq method produced larger insert sizes, higher alignment rates and higher library complexity with lower duplication rate as well as higher CpG coverage (39).

Although currently there are no standardization procedures for the routine clinical application of ctDNA/cfDNA analysis, several studies have highlighted the significance of variability and integrity of samples to ensure high-quality molecular tests (40–43). The pipeline gemBS used for processing EM-seq reads, enables the analysis of methylated DNA and CNVs, as well as single nucleotide variants, but not rearrangements. Nonetheless, single nucleotide variants were not the focus in this study. Our results reveal that evaluation of TapeStation electropherogram profiles offers a reliable approach to discriminate ctDNA from normal cfDNA. In our studies, 0.1 was established as the minimal ratio of ctDNA to normal cfDNA, ensuring that the tumor sample is sufficiently represented to perform subsequent methylome analysis. This is a crucial step for selecting samples that contain adequate ctDNA/normal cfDNA. A recent report suggests that total cfDNA concentrations in blood plasma from patients with high-risk neuroblastoma were higher than in healthy controls (44). However, in our cohort, 13 samples with apparently high concentration of cfDNA were classified as normal cfDNA or highly diluted ctDNA (i.e. patient TI-0572 with *MYCN* amplification at diagnosis had only 3 copies at relapse, with no other genetic or epigenetic alterations) after sequencing analysis. In healthy individuals, cfDNA is very low, averaging 10 to 15 ng per milliliter (45). In oncological patients, ctDNA can fluctuate from <0.1% to >90% of the cfDNA (46). We noted that cfDNA samples containing useful ctDNA (as determined by CNVs, hypomethylation profiles and PCA clustering) are enriched in mono-nucleosomal fragments (100-200 bp). In contrast, the presence of increasing amounts of multi-nucleosomal peaks (around 600 and 1000 bp) corresponding to four to six nucleosomes suggest dilution by normal cfDNA, except when high molecular weight DNA is present that may be secreted by tumor cells *via* alternative cellular processes. These distinct nucleosomal profiles facilitate identification of samples with high tumoral content which are appropriate for subsequent genomics procedures.

The origin of ctDNA is thought to be dependent on three cellular processes: apoptosis, necrosis and active cellular secretion (17). These different mechanisms by which ctDNA is liberated may contribute to the patient-specific differences and the fluctuating profiles that we observed in the electropherograms. In contrast to the low range of the apoptotic DNA ladder, necrosis is thought to generate ctDNA fragments generally larger than 10,000 bp, which is out of the range of detection by the TapeStation chips but can be quantified by other quality control techniques such as Qubit (**Table 3**). Interestingly, cfDNA fragments in the range of 1,000–3,000 bp are associated with active cellular secretion processes (47), and thus, these fragments may also be secreted by healthy cells (i.e. white cells in the blood) as a result of specific conditions of the patient. Tumor-specific alterations have been identified and monitored during disease progression in liquid biopsies from pediatric patients with high-risk neuroblastoma (44), as well as in adult tumors such as endometrial cancer (48), follicular lymphoma (49), non-small cell lung cancer (50), breast cancer (51), among others (reviewed in (52). Our results in high-risk neuroblastoma patients provide support for the early evaluation of valuable ctDNA content in liquid biopsy samples and represent an approach that could be extrapolated to other oncological patients.

CNV analysis derived from whole-genome methyl sequencing indicated that ctDNA was diluted with normal cfDNA in 13 samples (i.e., patient TI-0572, as previously discussed). In addition, the hypomethylation profile also enabled us to discriminate between tumor and normal cfDNA containing samples. DNA methylation changes are similar in most cells of a tumor but in contrast, the manifestations of gene mutations are more heterogeneous within the same tumor (53). Thus, the abnormal distribution of DNA methylation in our neuroblastoma patients, characterized mainly by a sharp methylation dropdown in discrete chromosomal regions, was also a useful tool to identify tumor profiles. The combination of ctDNA hypomethylation with CNVs analysis for further enhancement of the detection of at least one type of aberration to define an abnormality, was previously shown to have a sensitivity of 87% with a specificity of 88% in hepatocellular carcinoma (54). Thus, our results extend the application of ctDNA analysis to the detection and monitoring of high-risk neuroblastoma.

One interesting observation from our studies is that relapsed samples usually contained lower amounts of ctDNA (referred as to mono-nucleosomal peak) per ml of blood than their paired samples at diagnosis. Consistent with this, ctDNA at relapse was more easily masked by normal cfDNA, even though all samples were carefully subjected to the same technical procedures. Although we do not yet have a clear explanation for these findings, one possibility is that high-risk neuroblastoma at relapse is a more disseminated disease associated with less hypoxia. Therefore, hypoxia-associated processes such as apoptosis and necrosis, are also reduced and thus, less ctDNA is released into the bloodstream than at diagnosis. Alternatively, neuroblastoma relapses are more frequently found in bone marrow, which could represent a barrier for liberating tumor DNA to the bloodstream.

Collectively, our results support the use of cytosine enzymatic conversion over chemical modification as an optimal method to study ctDNA methylome with as little as 10 ng of DNA and provide a conceptual tool for the electropherogram-based detection of samples with useful ctDNA content for subsequent studies.

## Data availability statement

The datasets presented in this study can be found in online repositories. The names of the repository/repositories and accession number(s) can be found in the article/**Supplementary Material**.

## Ethics statement

La Fe Research Ethics Committee approved this project, reference number 2018-02-09. Written informed consent to participate in this study was provided by the participants’ legal guardian/next of kin.

## Author contributions

ET: Conception and design, collection and/or assembly of data, data analysis and interpretation, manuscript writing. AJ-R, GP, MG: Collection and/or assembly of data, data analysis and interpretation. VC, AC: Conception and design data analysis and interpretation, manuscript writing. SH: Conception and design data analysis and interpretation, manuscript writing. JF: Financial support, conception and design, collection and/or assembly of data, data analysis and interpretation, manuscript writing, coordination. All authors contributed to the article and approved the submitted version.
