# Supplementary figures and images for: Evaluation of circulating tumor DNA by electropherogram analysis and methylome profiling in high-risk neuroblastomas
Source: Front Oncol. 2023 May 12;13:1037342. doi: 10.3389/fonc.2023.1037342 (PMC10213460; doi:10.3389/fonc.2023.1037342)

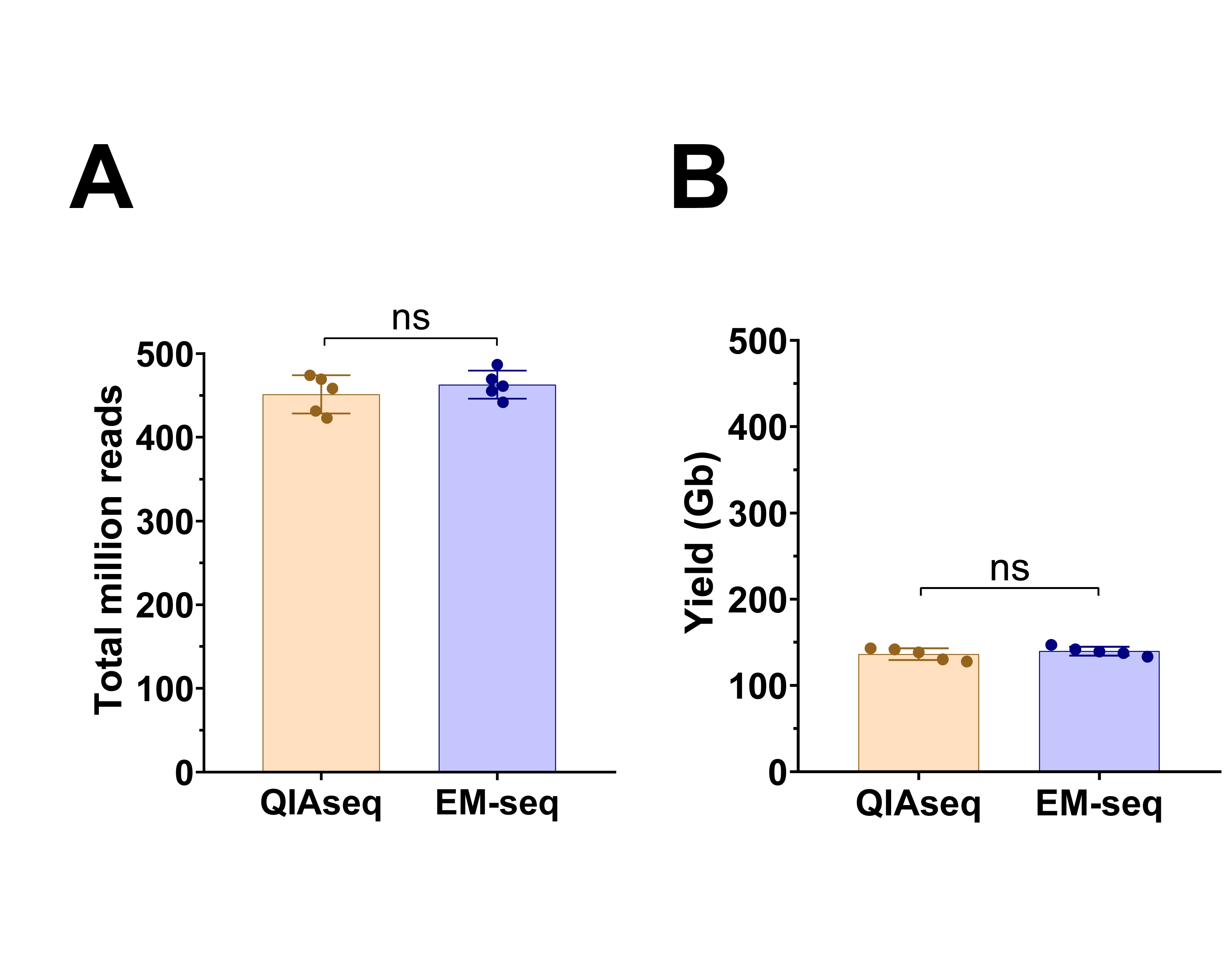

Supplement: Supplementary file 1 [file Image_1.tif]

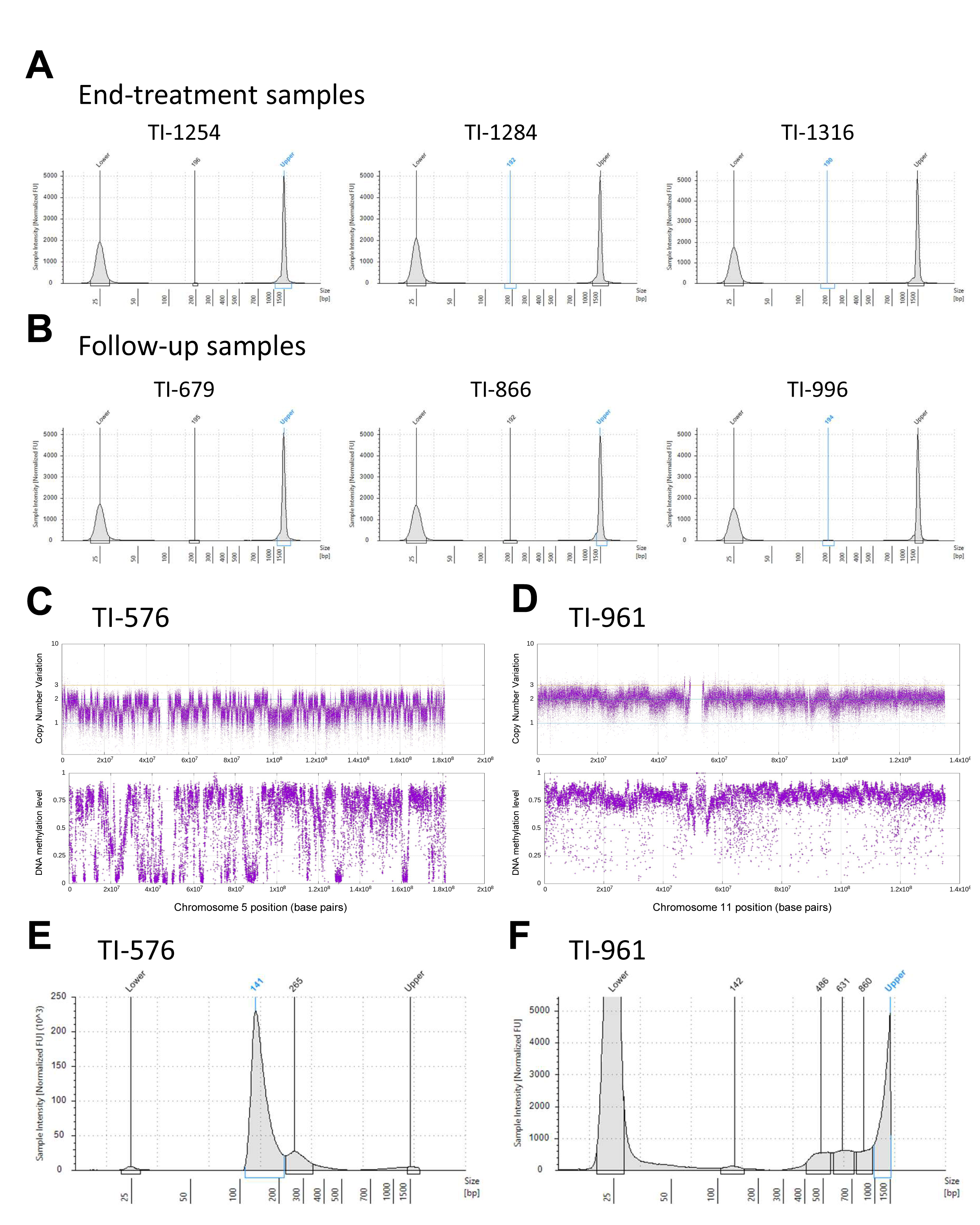

Supplement: Supplementary file 2 [file Image_2.tif]
